# Cross mapping of self-care interventions for expert patients

**DOI:** 10.17533/udea.iee.v42n3e08

**Published:** 2024-10-19

**Authors:** Manacés dos Santos Bezerril, Flávia Barreto Tavares Chiavone, Larissa Arielly Cunha da Silva, Isabelle Katherinne Fernandes Costa, Francisca Sánchez Ayllón, Maria Soledad Vázquez Santiago, Isabelle Campos de Azevedo, Viviane Euzébia Pereira Santos

**Affiliations:** 1 . Enfermeiro. Doutor. Email: manacesbezerril@hotmail.com https://orcid.org/0000-0002-9003-2334 Universidade Federal do Rio Grande do Norte Brazil manacesbezerril@hotmail.com; 2 . Enfermeira. Doutora. Email: flavia_tavares@hotmail.com . https://orcid.org/0000-0002-7113-2356 Universidade Federal do Rio Grande do Norte Brazil flavia_tavares@hotmail.com; 3 . Enfermeira. Mestre. Email: larissarielly@hotmail.com. Corresponding author. https://orcid.org/0000-0002-1929-2272 Universidade Federal do Rio Grande do Norte Brazil larissarielly@hotmail.com; 4 . Enfermeira. Doutora. Professora. Email: Isabelle.fernandes@ufrn.br. https://orcid.org/0000-0002-1476-8702 Universidade Federal do Rio Grande do Norte Brazil Isabelle.fernandes@ufrn.br; 5 . Enfermeira. Doutora. Professora. Email: fsayllon@ucam.edu https://orcid.org/0000-0003-2097-4147 Universidade Federal do Rio Grande do Norte Brazil fsayllon@ucam.edu; 6 . Enfermeira. Doutora. Professora. Email: svazquez@us.es. https://orcid.org/0000-0003-0607-330X Universidade Federal do Rio Grande do Norte Brazil svazquez@us.es; 7 . Enfermeira. Doutora. Professora. Email: isabellebr2511@gmail.com https://orcid.org/0000-0001-5322-7987 Universidade Federal do Rio Grande do Norte Brazil isabellebr2511@gmail.com; 8. Enfermeira. Doutora. Professora. Email: vivianeepsantos@gmail.com Universidade Federal do Rio Grande do Norte Brazil vivianeepsantos@gmail.com; 9 . Universidade Federal do Rio Grande do Norte, Brazil. Universidade Federal do Rio Grande do Norte Universidade Federal do Rio Grande do Norte Brazil

**Keywords:** self-care, standardized terminology in nursing, patient safety, nursing care, patient participation., autocuidado, terminología estandarizada en enfermería, seguridad del paciente, atención de enfermería, participación del paciente, autocuidado, terminologia padronizada em enfermagem, segurança do paciente, cuidados de enfermagem, participação do paciente.

## Abstract

**Objective.:**

To compare the primary nursing interventions described in the literature to promote self-care among expert patients with the Nursing Interventions Classification.

**Methods.:**

This descriptive and exploratory study used a quantitative approach and cross-mapping focusing on 23 self-care actions obtained from a scoping review conducted in January 2022 in Brazilian and international databases. Data were descriptively analyzed, processed, and presented in tables.

**Results.:**

Twenty-three self-care actions were found in the scoping review. A total of 56 interventions were selected, 30 of which were associated with self-care actions; some were associated with more than one intervention (e.g., physical activity, avoiding alcohol consumption, blood glucose monitoring, blood sugar management, stress/anxiety).

**Conclusion.:**

The self-care interventions promoted among expert patients were compared to the Nursing Interventions Classification, enabling nurses to lead by encouraging, assisting, teaching, discussing, and guiding patients. Hence, nursing workers can improve their work process by encouraging patient self-care and autonomy in the health-illness continuum.

## Resumen

**Objetivo.:**

Comparar las principales intervenciones de enfermería para el autocuidado del paciente experto descritas en la literatura con la Clasificación de Intervenciones de Enfermería.

**Métodos.:**

Estudio descriptivo, exploratorio, de enfoque cuantitativo con mapeo cruzado que se centró en 23 acciones de autocuidado, surgido de una revisión de alcance realizada en enero de 2022 en bases de datos nacionales e internacionales. Los datos fueron analizados, procesados ​​y presentados de forma descriptiva presentando los resultados en tablas.

**Resultados.:**

En la revisión del alcance se encontraron 23 acciones de autocuidado. Se seleccionaron 56 intervenciones, de las cuales 30 estuvieron asociadas a acciones de autocuidado, por lo que algunas de las acciones tuvieron más de una intervención, como actividad física, evitar el consumo de alcohol, monitoreo de glucemia, manejo de la glucemia, estrés/ansiedad.

**Conclusión.:**

Las intervenciones de autocuidado de pacientes expertos frente a la Clasificación de Intervenciones de Enfermería otorgaron al enfermero protagonismo en acciones para estimular, asistir, enseñar y orientar al paciente. Con esto, la enfermería mejora su proceso de trabajo fomentando el autocuidado y la autonomía del paciente en su proceso salud-enfermedad.

## Resumo

**Objetivo.:**

Comparar as principais intervenções de enfermagem para o autocuidado do paciente *expert* descritos na literatura com a Classificação das Intervenções de Enfermagem.

**Métodos.:**

Estudo descritivo, exploratório, de abordagem quantitativa com mapeamento cruzado que incidiu sobre 23 ações de autocuidado, oriundas de uma revisão de escopo realizada em janeiro de 2022 em bases de dados nacionais e internacionais. Os dados foram analisados, tratados e apresentados de maneira descritiva mediante apresentação dos resultados em tabelas.

**Resultados.:**

Foram encontradas 23 ações de autocuidado na revisão de escopo. Selecionaram-se 56 intervenções, das quais, 30 foram associadas com as ações de autocuidado, de modo que algumas das ações dispunham de mais de uma intervenção, como a exemplo de atividade física, evitar o consumo de álcool, monitoramento da glicemia, manejo do estresse/ansiedade.

**Conclusão.:**

as intervenções de autocuidado dos pacientes *experts* comparadas a Classificação das Intervenções de Enfermagem, proporcionam ao enfermeiro o protagonismo de ações para estimular, auxiliar, ensinar, discutir e orientar o paciente. Com isso, a enfermagem melhora seu processo de trabalho ao estimular o autocuidado e a autonomia do paciente em seu processo de saúde-doença.

## Introduction

The term expert patient concerns individuals who can seek information on health topics like diseases, diagnoses, medications, symptoms, and treatments for themselves and others, as well as discuss such aspects with health professionals. Thus, the expression emerged to refer to an active, self-confident individual able to manage his/her care.^1^^-^^3^ The expression patient expert appeared in 1990 at Stanford University, with the “Chronic Disease Self-Management Program,” aimed to transfer/share knowledge and skills related to the care required by a disease. This methodology enabled the program to be expanded worldwide, starting with the first schools opened in European countries. For example, the Expert Patient Programme (EPP) was proposed in 2002 in the United Kingdom and is now adopted in other parts of the world to develop and implement new management policies for people with chronic diseases.^1^^,^^4^ Most expert patients have at least one chronic disease. They are trained to become protagonists of their care, capable of establishing strategies and making decisions to achieve positive results in their treatment.^5^

Chronic diseases are a common global health problem, imposing high costs on the healthcare sector. Therefore, strengthening information technology support systems aimed at patient- and family-centered care is essential, as well as involving professionals from both levels of care.^6^ Note that the participation of health professionals in this process builds a horizontal relationship and enables the establishment of ties of trust to achieve positive results in patient care. In this sense, nursing workers stand out because they are present at different levels of complexity and seek measures to intervene in the health-disease continuum to promote care.^2^^,^^7^ Therefore, taxonomies are one of the strategies that can promote a standardized language in nursing care, enabling the comparison and evaluate the care provided in different contexts by different professionals. At the same time, the Nursing Interventions Classification (NIC) emerged as a clinical tool with standardized language that describes the treatments nurses implement among patients, supports clinical decision-making, assists managers in personnel and equipment dimensioning, and facilitates communication, among other benefits that contribute to the advancement of care.^8^

This widely adopted system is part of the operationalization of nursing care based on the Nursing Process (NP). This methodological instrument guides the care and practice of the nursing team. The NP is organized into five interrelated and interdependent stages: data collection, nursing history investigation, diagnosis, planning, implementation, and evaluation.^9^

Thus, using NIC to manage the care of expert patients can promote greater independence and safety for the self-management of their clinical conditions.^8^ Additionally, interventions must adapt to the patient’s particularities so that they can become active agents managing self-care, complementing the educational work performed by healthcare professionals.^1^^,^^2^ Thus, the question guiding this study is: Which of the expert patients’ primary self-care interventions are related to NIC taxonomies? The objective is to compare the primary nursing interventions for the self-care of expert patients described in the literature with NIC. 

## Methods

This descriptive, exploratory, cross-mapping study adopted a quantitative approach to compare the interventions listed in the scientific literature on self-care actions for expert patients according to the taxonomy of standardized nursing interventions NIC.^10^^,^^11^ Thus, the cross-mapping method was used as a technique for analyzing non-standardized nursing languages compared to a standardized nursing language.^11^ Three adapted steps were applied to use the cross-mapping method: 1) an analysis of the expert patients’ self-care; 2) the NIC nursing interventions/activities were selected for each self-care action listed; 3) a list of NIC interventions/activities for each self-care listed.^11^ Furthermore, six rules must be considered in the mapping: 1) Selecting a NIC for each self-care listed; 2) Selecting a NIC based on the similarity between self-care and the establishment of the intervention and suggested activities; 3) Determining a keyword from the self-care list, which will help identify the most appropriate intervention/activity in the NIC; 4) Preferably using the verbs used in self-care to select the NIC/activity; 5) Mapping the self-care that uses two verbs, in two different NIC interventions, when the actions are different; 6) Identifying and describing the self-care that cannot be mapped, for any reason.^11^ Hence, the cross-mapping focused on 23 self-care actions obtained in a scoping review conducted in January 2022, according to the Joanna Briggs Institute Reviewer’s Manual proposed by the theoretical model according to Arksey and O’Malley, based on the Preferred Reporting Items for Systematic reviews and Meta-Analyses extension for Scoping Reviews (PRISMA-ScR): Checklist and Explanation, and research protocol registered in the Open Science Framework (OSF), DOI 10.17605/OSF.IO/YPUVM.^12^^-^^14^

The following descriptors and keywords were combined to search for the self-care actions: [Chronic Disease OR (Chronic Cases OR Chronic Pictures OR Chronic illness OR Degenerative disease) AND Self Care OR (Self Help OR Self-management of care) AND Primary Health Care OR (Primary Care OR Primary attention OR Basic Service)], which resulted in a final sample of 234 studies. Note that the term expert patient was not used in the search strategy because it is not a controlled descriptor but refers to an individual with a chronic disease.

The following databases were consulted: U.S. National Library of Medicine (PubMed) and Cumulative Index to Nursing and Allied Health Literature (CINAHL), SCOPUS, Cochrane CENTRAL, Web of Science, PsycINFO, Latin American and Caribbean Literature in Health Sciences (LILACS) and Educational Resources Information Center (ERIC), CAPES Theses and Dissertations, the National Library of Australia’s Trobe (Trove), Academic Archive Online (DIVA), DART-Europe E-Theses Portal, Electronic Theses Online Service (EThOS), Open Access Scientific Repository of Portugal (RCAAP), National ETD Portal, Theses Canada, Theses and Dissertations from Latin America. Based on the findings regarding self-care actions, the evaluators formed pairs to cross-map the self-care actions performed by expert patients found in the scientific literature and the nursing interventions indexed in the NIC. A third evaluator resolved potential divergences between the two evaluators regarding mapping self-care actions and NIC interventions.

Descriptive statistics were used to analyze, process, and present the results in tables. Furthermore, as this study addresses information in the public domain and does not involve human subjects, there was no need for it to be assessed by an Institutional Review Committee. 

## Results

Of the 23 self-care actions found in the scoping review, only acupuncture did not integrate the cross-mapping, as no intervention indexed in the NIC was found that was directly related to this practice. Regarding the cross-mapping (Table 1), 56 interventions were first selected. However, the reviewers associated 30 of these with self-care actions, so some of the actions are associated with more than one intervention, such as physical exercise, avoiding alcohol consumption, blood glucose monitoring, stress/anxiety management, hygiene/appearance, pain management, breathing exercises, and cognitive exercises/activities. The activities chosen for each NIC intervention were based on the use of verbs or sentences that convey the idea that the nurse contributes to promoting the patient’s active role in their clinical care, such as teaching, encouraging, assisting the patient, discussing with the patient, encouraging, explaining, and guiding, among others. Additionally, such activities may be indicated and adapted for more than one chronic disease and different expert patient profiles (age, culture, gender, level of education, family income, among others).

**Table 1:** Cross-mapping between self-care actions performed by expert patients found in the scoping review with the interventions and activities in the Nursing Interventions Classification (NIC).

Self-care actions	NIC	NIC Activities
Physical activity	EXERCISE promotion: Strength training (0201)	Helping the patient to express his/her beliefs, values, and goals for achieving muscle fitness and health. Helping the patient establish realistic goals in the short and long terms and engage with the exercise plan. Helping the patient to create an environment at home/workplace that facilitates engaging with the exercise plan. Helping the patient devise a continuous recording system that includes the gym weights and the number of repetitions and sequences to monitor progress in muscle fitness.
	EXERCISE therapy: control muscle control (0226)	Explaining why specific exercises are chosen and the protocol established for the patient/family Helping the patient establish realistic and measurable goals. Incorporating activities of daily life into the exercise protocol, if appropriate. Encouraging the patient to exercise independently, if indicated.
Balanced diet	EATING Disorders Management (1030)	Establishing expectations toward adequate eating behavior, food and fluid intake, and physical exercise. Holding the patient responsible for his/her diet choices and physical activity, as appropriate. Helping the patient to assess how adequate his/her choices regarding diet and physical activity are and their respective consequences.
Medication adherence	TEACHING: prescribed medication (5616)	Teaching the patient to recognize the medications’ different characteristics as appropriate. Teaching the patient about the purpose and action of each medication. Teaching the patient about the dosage, route, and duration of each medication. Teaching the patient about the administration/correct application of each medication. Revise what the patient knows about medications. Teaching the patient to perform the necessary procedures before taking medications, as appropriate. Informing the patient about the consequences of not taking the medication or stopping it suddenly, as appropriate. Informing the patient about potential drug/food interactions, as appropriate. Teaching the patient about the correct disposal of needles and syringes at home and where to place the sharps container in the community, as appropriate. Helping the patient to organize a written medication schedule. Warn the patient about giving prescribed medications to other people. Provide information about programs/organizations to save financial resources when buying medications and devices, as appropriate. Providing information about medication alert devices and how to obtain them.
Regular consultations/exams	Telephone CONSULTATION (8180)	Considering cultural and socioeconomic barriers regarding the patient’s reactions. Identifying the patient’s concerns regarding health status. Establishing the knowledge of the person on the telephone and the source of such knowledge. Determining the patient’s ability to understand teaching/instructions over the phone. Inform the patient about prescribed therapies and medications, as appropriate. Identifying actual/potential problems regarding the self-care regime. Involving the family/significant persons in the care and planning. Answering questions.
Social activities	SOCIALIZATION Enhancement (5100)	Encouraging patients to improve their involvement in already-established relationships. Encouraging patients to be tolerant when developing relationships. Promoting relationships with people who share common interests and goals. Encouraging social and community activities. Encouraging patients to share common problems. Encouraging patients to become involved with completely new interests. Encouraging patients to participate in group or individual remembrance activities. Facilitating the patient’s participation in groups that tell stories. Helping the patient to improve perception of his/her communication strengths and weaknesses. Encouraging the patient to change environments, such as going for a walk or to the movies. Encouraging the patient to plan special activities in small groups.
Avoiding alcohol consumption	SUBSTANCE use treatment (4510)	Identifying factors (e.g., genetic, psychological distress, stress) contributing to chemical dependency. Encouraging the patient to take control over his/her behavior. Helping patient/family to identify denial as a substitute for confronting the problem. Helping the patient identify the adverse effects of chemical dependency on health, family, and daily functioning. Identifying with the patient constructive goals to offer alternatives to the use of substances to alleviate stress. Helping the patient to choose an alternative activity that is compatible with the abused substance. Identifying support groups in the community to treat long-term substance abuse.
	SUBSTANCE use prevention (4500)	Helping the person tolerate higher stress levels, as appropriate. Encouraging responsible decision-making regarding lifestyle options. Helping the patient to identify strategies to decrease tension.
Avoiding smoking	SMOKING Cessation Assistance (4490)	Determining the patient’s readiness to learn how to quit smoking. Giving clear and coherent advice to quit smoking. Helping the patient to identify the reasons and barriers to quitting smoking. Reassuring the patient that nicotine withdrawal symptoms are temporary. Helping the patient identify psychosocial aspects influencing a smoker’s behavior. Helping the patient devise a plan to quit smoking that addresses psychosocial aspects influencing a smoker’s behavior. Helping the patient to recognize indicators that lead him/her to smoke. Helping the patient to develop practical methods to resist strong desires. Encouraging the patient to keep a cigarette-free lifestyle. Encouraging the patient to join a smoking cessation support group that s/he meets weekly. Helping the patient with self-help methods.
Hyperglycemia monitoring	HYPERGLYCEMIA management (2120)	Monitoring for hyperglycemia signs and symptoms: polyuria, polydipsia, polyphagia, weakness, lethargy, malaise, blurred vision, or headache. Encouraging oral intake of fluids. Monitoring fluid status (including intake and output) as appropriate. Instructing the patient and significant others on how to prevent, recognize, and manage hyperglycemia. Encouraging self-monitoring of blood glucose levels. Helping the patient to interpret blood glucose levels. Facilitating the patient to adhere to the diet and exercise regimens.
	HYPOGLYCEMIA control (2130)	Monitoring hypoglycemia signs and symptoms. Providing feedback regarding adequate hypoglycemia self-control. Instructing the patient and significant others on hypoglycemia signs and symptoms, risk factors, and treatment. Encouraging self-monitoring of blood glucose levels.
Foot care	FOOT care (1660)	Examining the skin for irritation, cracks, lesions, bunions, calluses, deformities, or edema. Carefully drying between the toes. Applying creams. Cleaning the nails. Discuss regular foot care routine with the patient. Instructing the patient/family on the importance of foot care. Instructing the patient to examine the inside of shoes for rough areas. Instructing the patient on the importance of inspections, especially when there is decreased sensation. Referring to a podiatrist for trimming thick toenails, as appropriate. Instructing the patient on how to prepare and trim toenails.
Regular sleep/ rest	SLEEP enhancement (1850)	Instructing the patient to monitor sleep patterns Encouraging the patient to establish a bedtime routine to facilitate the transition from wakefulness to sleep. Instructing the patient to avoid foods and beverages at bedtime that may interfere with sleep. Helping the patient limit daytime sleep by providing activities that promote wakefulness, as appropriate. Instructing the patient to use autogenic muscle relaxation or other nonpharmacological forms of sleep induction.
Weight management	WEIGHT management (1260)	Discuss with the patient the relationship between food intake, exercise, weight gain, and weight loss. Discuss with the patient the habits, customs, and cultural and hereditary factors influencing weight. Discussing the risks associated with being overweight or underweight. Determining the individual's motivation to change eating habits. Developing with the individual a method of keeping a daily record of food intake, exercise sessions, and changes in body weight. Encouraging the individual to write down realistic weekly goals for good food intake and exercise and display them where they can be reviewed daily. Encouraging the individual to record his/her weight every week on a chart, as appropriate. Encouraging the individual to consume adequate amounts of water daily.
Stress/ anxiety management	RESILIENCY promotion (8340)	Encouraging family/community to value achievements. Encouraging family/community to value health. Encouraging positive health-seeking behaviors. Facilitating the development and use of neighborhood resources.
	EMOTIONAL support (5270)	Talking with the patient about the emotional experience(s). Exploring with the patient what triggered the emotions. Helping the patient identify feelings, such as anxiety, anger, or sadness. Encouraging the patient to express feelings of anxiety, anger, or sadness. Encouraging talking or crying as a way to decrease emotional response. Referring for counseling, as appropriate
Hygiene/ Appearance	SELF-CARE assistance: bathing/hygiene (1801)	Considering the patient's culture when promoting self-care activities. Considering the patient's age when promoting self-care activities. Providing care until the patient is fully capable of assuming self-care. Helping the patient determine the extent of actual changes in the body or level of functioning.
	Body IMAGE enhancement (5220)	Helping the patient separate physical appearance from feelings of self-worth, as appropriate. Helping the patient determine the peer influence on his/her perception of current body image. Facilitating contact with people experiencing similar changes in body image. Identifying support groups
Goal setting	Mutual GOAL setting (4410)	Encouraging the patient to identify specific life values. Determining how the patient recognizes the problem. Encouraging the patient to identify his/her strengths and capabilities. Helping the patient identify realistic and achievable goals. Developing and using a goal attainment scale, as appropriate. Identifying goals together with the patient. Assisting the patient in assessing the resources available to achieve the goals. Helping the patient establish a realistic timeline. Coordinating with the patient periodic review dates to assess progress toward goals. Reevaluating goals and the plan, as appropriate.
Blood pressure measurement	VITAL SIGNS monitoring (6680)	Monitoring blood pressure, pulse, temperature, and respiratory pattern as appropriate Checking apical and radial pulses simultaneously and note differences as appropriate. Monitoring skin color, temperature, and moisture. Identifying potential causes for vital signs changes. Periodically checking the accuracy of the instruments used to obtain patient data.
Pain management	Patient-controlled ANALGESIA (PCA) assistance (2400)	Teaching the patient and family to monitor pain intensity, quality, and duration. Teaching the patient and family to monitor respiratory rate and blood pressure. Teaching the patient and family to use the PCA device. Teaching the patient and family to adjust the infusion rate to the appropriate level on the PCA device. Teaching the patient and family about pain-relieving agents’ actions and side effects.
	PAIN management (1400)	Investigate the patient's knowledge and beliefs about pain. Investigate with the patient the factors that alleviate/worsen the pain. Teaching pain management principles. Encouraging the patient to monitor his/her pain and intervene appropriately. Teaching nonpharmacological techniques before, during, and after painful activities, whenever possible, before pain occurs or worsens, and in conjunction with other pain relief measures. Encouraging the patient to talk about his/her pain experience, as appropriate. Incorporating the family into the pain relief method whenever possible.
Meditation	MEDITATION facilitation (5960)	Instruct the patient to sit comfortably. Instruct the patient to close his/her eyes, if desired. Instruct the patient to relax all muscles and remain relaxed. Helping the patient choose a mental resource to be repeated during the procedure. Instruct the patient to mentally/silently repeat the resource while exhaling through the nose. Instruct the patient to ignore scattered thoughts and return to the mental resource. Instruct the patient to perform the procedure once or twice a day but wait two hours after meals.
Breathing exercises	RESPIRATORY monitoring (3350)	Monitoring breathing frequency, rhythm, depth, and effort.
	VITAL SIGNS monitoring (6680)	Monitoring blood pressure, pulse, temperature, and respiratory pattern, as appropriate. Monitoring respiratory rate and rhythm (e.g., chest depth and symmetry). Identifying potential causes of changes in vital signs.
Daily inhalation	MEDICATION administration: inhalation (2311)	Determining the patient's knowledge of the medication and understanding of the method of administration. Instruct the patient to tilt the head back slightly and exhale completely. Instruct the patient to repeat inhalations as recommended, waiting at least one minute between inhalations. Teaching and monitoring the self-administration technique, as appropriate.
Adequate water intake	FLUID management (4120)	Monitoring hydration status (e.g., moist mucous membranes, adequate pulse, and orthostatic blood pressure) as appropriate. Monitoring food/fluid intake and calculating daily caloric intake as appropriate. Encouraging oral intake
Cognitive exercises/ activities	MEMORY TRAINING (4760)	Implementing appropriate memorization techniques, such as visual images, mnemonic resources, memory games, memory indicators, association techniques, adopting lists, using a computer, labels, or information rehearsal. Assisting associated learning tasks such as learning by doing and recalling verbal and pictorial information as appropriate. Providing training and guidance, such as rehearsal of personal information and dates, as appropriate. Providing guidance to the patient to acquire new learning, such as locating geographical aspects on a map, as appropriate. Providing recognition memory of photographs/pictures, as appropriate. Encouraging the patient to participate in group programs to train memory, as appropriate.
	COGNITIVE stimulation (4720)	Talking to the patient. Asking the patient to repeat information. Giving verbal and written instructions.
Safe sex	TEACHING: safe sex (5622)	Counseling the patient to use effective birth control methods as appropriate Encouraging the patient to be selective when choosing sexual partners, as appropriate. Counseling the patient on low-risk sexual practices, such as those that avoid penetration or exchange of body fluids, as appropriate. Reinforcing the use of condoms. Encouraging patients at high risk for sexually transmitted diseases to have regular checkups.

## Discussion

The diversity of self-care actions available to expert patients indicates an increase in ways patients can be encouraged to take an active role in their care to achieve positive results, considering that many of these activities can be included or adapted for more than one chronic disease, in the case of patients with comorbidities.^15^^-^^17^ Thus, nursing workers have the opportunity to improve the work process by planning, together with expert patients, the self-care actions that will be implemented according to each patient’s particularities, considering daily routine, behaviors, family and social environment, feelings, culture, monthly income, experiences, and perceptions, as these are directly related to how a patient sees and deals with his/her clinical condition.^15^^-^^20^

Furthermore, Achury-Saldaña *et al*.,^1^ Bastos *et al*.,^7^ Freilich *et al*.,^15^ and Robles-Sanchez *et al*.^20^ state that encouraging self-management of care is a strategy for expanding nursing care beyond the physical sphere of health services, considering improved health teaching practices, the fact that the nursing profession has been increasingly appreciated, a recognition of the tools developed and used by nurses, and improved patient-professional relationships.^1^^,^^7^^,^^15^^,^^20^ Therefore, the intersection of self-care actions obtained in the scoping review on the NIC/activities shows that nurses can encourage expert patients to become autonomous and perform proper self-care, a practice implemented even before this new patient profile was disseminated and understood; the taxonomy of nursing interventions was developed and began being used in the 1980s, while the term expert patient emerged ten years later, precisely when the number of people living with a chronic disease grew exponentially worldwide.^2^^-^^4^^,^^8^^,^^21^^,^^22^ Furthermore, NIC is constantly updated, and new interventions/activities are added to meet global and epidemiological transformations,^8^ which possibly explains why some self-care actions have more than one corresponding NIC intervention, among which the following stand out: physical activity, blood glucose monitoring, stress/anxiety management, breathing exercises, and cognitive exercises/activities.

The self-care actions listed above lead to a greater depth of clinical knowledge, improving patient outcomes and providing a source of data for future research. Thus, it is important to note the importance of activities intended to monitor, prevent, and resolve potential clinical problems that may affect a patient’s treatment and quality of life.^1^^,^^4^^,^^9^ Note that these self-care practices are directly associated with diseases such as obesity, diabetes, chronic obstructive pulmonary disease, depression, Alzheimer’s, and anxiety, which are part of the group of chronic diseases most frequent in recent years.^17^^,^^21^^,^^22^

Thus, Rocha *et al.*^23^ and Soares-Pinto *et al.*^24^ agree that there should be an incentive to conduct scientific research more focused on self-care actions that can be inserted, clarified, and optimized in the NIC to cover the growing and diverse number of chronic diseases affecting the population requiring devices to assist in their independent clinical management, including the possibility of solving daily problems, without the need to commute to a healthcare facility.^23^^,^^24^ Among the actions mapped, the nursing team involved in this process stands out as the agents who assist in such activities to provide and regulate direct care delivery in patients’ daily routines, meeting social, educational, and care needs. In this sense, the bio-psycho-social-spiritual human being needs to adapt to his/her environment to maintain balance and good behavior in the dimensions proposed. In this sense, nurses are educators who perform these activities pertinent to the profession.^25^

In this regard, the NIC interventions, combined with the self-care actions presented in the scoping review, revealed a strong connection with the care that nurses promote in hospital settings. Such connection may lead to a perception disconnected from the concept of expert patient and support that prioritizes self-care management beyond the scope of health services. Nevertheless, although the activities linked to the interventions are performed by nursing workers, the interventions selected in this study are those aimed at promoting self-care, as they enable nurses to assess, together with their patients, their knowledge and understanding of their conditions, limitations, as well as those of their families within the context of their homes, and the possibilities available for adapting care.^1^^,^^9^^,^^19^^,^^23^ However, encouraging an individual to become an active agent responsible for his/her self-care does not make one an expert patient, as such knowledge requires a detailed assessment of favorable aspects, followed by complementary training.^2^^,^^4^^,^^5^^,^^20^ Another aspect to highlight is the absence of a NIC intervention linked to the acupuncture self-care action. The reason, according to Bousfield *et al*.^26^ and Maul *et al*.,^27^ is that, despite an increased appreciation and implementation of complementary therapies originating from Eastern medicine in Western health services given the many positive results, there are few activities with this profile in the NIC taxonomy. Hence, future studies are suggested to investigate similar practices to be considered and implemented according to self-care management.^26^^,^^27^ Thus, there are many self-care activities nurses can or should implement to meet the diverse representations of expert patients, as these individuals are increasingly able to deal with their clinical condition through actions that can be adjusted and adapted.

Conclusion. Among the primary self-care interventions promoted among expert patients related to the NIC taxonomies, the following stand out: physical activity, balanced diet, medication adherence/use, regular consultations/examinations, social activities, avoiding alcohol and tobacco consumption, and blood glucose monitoring. These activities implemented in interventions enable nurses to encourage, assist, teach, discuss, and guide patients. Therefore, nurses improve their work process by encouraging patient self-care and autonomy to deal with the health-disease continuum. Furthermore, despite a cross-mapping based on nursing interventions for self-care actions obtained from a scoping review, information supports the transformation, improvement/reconstruction of a language used by nurses in the work process directed at expert patients. However, one of this study’s limitations concerns the strong reference in the NIC to nursing care provided in the hospital setting. Such a reference contradicts what is proposed for expert patients in primary health care. Therefore, further studies are needed to address activities that prioritize these patients’ self-care management in other healthcare settings. Therefore, promoting research, discussions, and the validation of new nursing taxonomies concerning self-care interventions for expert patients and disseminating them is essential to strengthening the care continuity for patients and nursing workers in different healthcare services.
